# Molecular Mechanisms and Treatment Strategies in Diabetic Nephropathy: New Avenues for Calcium Dobesilate—Free Radical Scavenger and Growth Factor Inhibition

**DOI:** 10.1155/2017/1909258

**Published:** 2017-09-26

**Authors:** Hermann Haller, Linong Ji, Klaus Stahl, Anna Bertram, Jan Menne

**Affiliations:** ^1^Department of Nephrology, Hannover Medical School, Carl-Neuberg-Str. 1, 30625 Hannover, Germany; ^2^Department of Endocrinology and Metabolism, Peking University People's Hospital, No. 11 Xizhimen South Street, Xicheng District, Beijing 100044, China

## Abstract

Diabetic nephropathy is one of the most important microvascular complications of diabetes mellitus and is responsible for 40–50% of all cases of end stage renal disease. The therapeutic strategies in diabetic nephropathy need to be targeted towards the pathophysiology of the disease. The earlier these therapeutic strategies can bring about positive effects on vascular changes and prevent the vasculature in patients with diabetes from deteriorating, the better the renal function can be preserved. Studies evaluating anti-inflammatory and antioxidative strategies in diabetic nephropathy demonstrate the need and value of these novel treatment avenues. CaD is an established vasoactive and angioprotective drug that has shown a unique, multitarget mode of action in several experimental studies and in different animal models of diabetic microvascular complications. On the molecular level, CaD reduces oxidative stress and inhibits growth factors such as fibroblast growth factor and vascular endothelial growth factors. Recent findings have demonstrated a strong rationale for its use in reducing urine albumin excretion rate and markers of inflammation as well as improving endothelial function. Its beneficial effects make it an attractive therapeutic compound especially in the early stages of the disease. These findings, although promising, need further confirmation in prospective clinical trials with CaD.

## 1. Introduction

Diabetes mellitus (DM) is a leading cause of morbidity and mortality. Vascular lesions from microvascular and macrovascular involvement lead to impaired blood flow and contribute to damage and dysfunction of one or more target organs, that is, the heart, kidneys, eyes, and nervous system. Diabetic nephropathy (DN) is one of the most important microvascular complications of DM and is responsible for 40–50% of all cases of end stage renal disease (ESRD) [1]. Among the patients with type 1 diabetes (T1D), as many as one-third develop serious renal complications, characterized by increasing urinary albumin excretion rates (AER) and decreasing kidney function, measured by eGFR, with 10%–20% of the subjects progressing to ESRD [1]. Diabetic nephropathy is therefore the leading cause of ESRD and when confounded by hypertension, it is the leading risk factor for cardiovascular disease [1].

## 2. Proteinuria and Diabetic Nephropathy

A marker of DN is albuminuria, arbitrarily defined as microalbuminuria (urine albumin excretion <300 mg/24 h) and macroalbuminuria (>300 mg/24 h). Compared to diabetic patients with normoalbuminuria (<30 mg/24 h), patients with persistent macroalbuminuria (overt DN) have an almost 10-fold higher risk of developing ESRD [2]. Histologically, DN manifests as diffuse or nodular mesangial expansion, tubular and glomerular basement membrane thickening, and interstitial fibrosis [3]. However, few diabetic patients presenting with albuminuria are biopsied. Therefore, the hallmark of DN diagnosis remains as urinary protein excretion.

However, recently it has become evident that in a subgroup of diabetic patients nephropathy can occur without going through a phase of proteinuria but rather show a rise in the prevalence of reduced renal function as measured by eGFR without significant proteinuria. Indeed, whereas diabetic nephropathy is traditionally defined as the presence of proteinuria or progression to ESRD, there is now increasing utilization of decreased renal function, as reflected by declined eGFR, in the definition of diabetic kidney complications.

The detection and diagnosis of DN can be a challenge due to its insidiousness. The onset of microalbuminuria is the earliest clinical manifestation of renal injury in most patients with diabetes [2]. Without specific interventions, many patients with DM progress from microalbuminuria to macroalbuminuria. The presence of macroalbuminuria especially when it is associated with a decrease in renal function (i.e., decrease in glomerular filtration rate [GFR]) is considered diagnostic for overt DN. Patients with type 1 DM generally present with microalbuminuria 6–10 years after being diagnosed with diabetes. Of these patients, 20–45% progress to DN during the next 10 years. Overt DN occurs therefore about 20 years after the diagnosis of type 1 DM in many patients [2, 4].

In type 2 DM, the natural history of DN development is similar but due to delayed diagnosis of their diabetes, type 2 DM patients may already have overt proteinuria and impaired GFR at the time of diagnosis (or shortly after their diabetes diagnosis). In these patients the duration of diabetes is often not precisely known. This makes it difficult to prevent and to treat DN in a timely manner. Renal endpoints, such as ESRD or doubling of serum creatinine, occur mostly within ten years in approximately 20% of microalbuminuric patients and in more than 60% of macroalbuminuric patients. The incidence of renal complications is more or less similar in type 1 and 2 DM [5–7].

The cellular and molecular mechanisms involved in the pathophysiology of proteinuria in diabetes are worth exploring when evaluating therapeutic strategies that interfere with the development and progression of DN. Hyperglycemia can lead to hemodynamic changes in the glomerulus, endothelial cell dysfunction, changes in the basal membrane, and podocyte injury. All of these mechanisms are likely involved in DN development at different time points. Therapeutic strategies, however, are often directed at one or two of these pathophysiological changes. The best example of this is the blockade of the renin-angiotensin system (RAAS) in DN. Glomerular hyperfiltration is present from the onset of diabetes until macroalbuminuria presents.* During the rise of macroalbuminuria GFR declines rapidly*. This sequence of events is consistent with the hypothesis that glomerular hyperfiltration causes progressive glomerular damage and that the subsequent pathological changes in the interstitium are mediated by microinflammation followed by interstitial fibrosis. Inhibition of the RAAS improves glomerular hyperfiltration by dilating the efferent glomerular arterioles thereby reducing glomerular pressure and proteinuria. In addition to these hemodynamic effects RAAS inhibition has distinct molecular effects on cell functions such as hypertrophy, proliferation, and migration. Inhibition of angiotensin II affects endothelial cells, mesangial cells, and podocytes. In this review, the pathological mechanisms of DN will be explored further, as well as the therapeutic role that CaD, a molecule with anti-inflammatory, antioxidative, and antiangiogenic properties, may play in DN.

## 3. Histology of Diabetic Nephropathy

The first reports from renal biopsies of patients with diabetic kidney disease described the initial changes of the disease, that is, glomerular hypertrophy, mild mesangial expansion with matrix accumulation, and thickening of the glomerular capillary walls [8]. These changes have also been consistently observed by electron microscopy. With progression of the disease, there is further increase in mesangial expansion with accumulation of extracellular material in the mesangium. In subsequent years the glomerular volume may increase further and there may be formation of hyaline nodules in the glomerular tuft. This histological pattern is referred to as nodular diabetic glomerulosclerosis or Kimmelstiel-Wilson disease [9, 10]. In the past, Kimmelstiel-Wilson has been used as a synonym of DN. The presence of nodules of different size, at times laminated, with variable distribution in the glomeruli, is pathognomonic of DN. From this stage of the disease, progressive thickening of capillary walls and global glomerulosclerosis is observed [8]. Two other typical glomerular lesions are (i) capsular drop, a homogeneous hyaline deposit in Bowman's capsule and (ii) glomerular hyalinosis [11–13]. However, these changes have also been observed in other glomerular diseases [14]. In typical cases of DN, microaneurysms, produced by mesangiolysis, are evident.

The changes in the tubulointerstitium are more consistent. In tubules, increased reabsorption of protein, interstitial fibrosis, followed by tubular atrophy is observed [8, 9]. Most likely, these changes are associated with activation of tubular cells and induction of inflammatory mechanisms. Macrophages invade the interstitium thus contributing to interstitial inflammation and fibrosis. Over time, the tubules disappear and are replaced by fibrotic tissue.

Blood vessels of the diabetic kidney are characterized by intimal hyaline thickening of arterioles and rarefication of capillaries [15, 16]. Arteriolar lesions may involve any of the arterioles. Intimal fibrosis of the arteries is also common in DN, but this feature is not pathognomonic because intimal fibrosis occurs in other diseases as well. The most important is the presence of activated and damaged endothelial cells in the diabetic kidney [17]. It is hypothesized that endothelial cells are damaged and respond by activation of inflammatory mechanisms, recruit inflammatory cells from the circulation, and contribute to the inflammatory state of the diabetic kidney [18]. Despite the increased knowledge about the pathophysiological mechanisms of diabetic kidney disease, it is still unclear how metabolic and hemodynamic damage to endothelial and epithelial cells, inflammation, and progressive fibrosis with deterioration of the microcirculation are connected. However, the prevention of microvascular damage and the inhibition of inflammatory mechanisms would appear to be important therapeutic and preventative goals in DN.

## 4. Pathogenesis of Diabetic Nephropathy: A Comprehensive View

**Figure 1:**
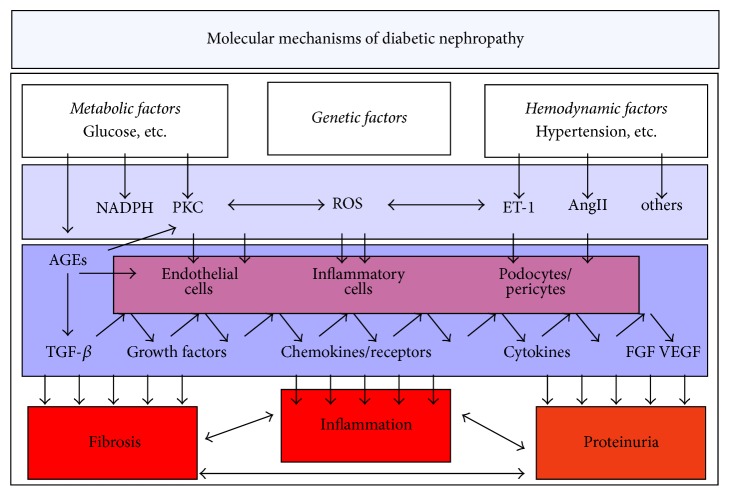
A schematic overview on structures and mechanisms in the pathogenesis of diabetic nephropathy. The pathogenesis of diabetic nephropathy involves several mechanisms over the course of the disease. Hyperglycemia is the leading cause of diabetic nephropathy; however, other metabolic factors such as hypercholesterolemia also play a role. Genetic factors are prominent since only ca. 30% of diabetic patients develop diabetic nephropathy. Importantly, hypertension and hemodynamic factors in the kidney, that is, hyperfiltration contribute significantly to the development of the disease. These external factors are translated by several intracellular pathways such as NADPH or PKC into cell activation. Different cell types respond in a specific fashion. Growth factors such as VEGF or TGF-b and chemokines such as MCP-1 are expressed and lead to inflammation and proteinuria followed by fibrosis. AngII, angiotensin II; ET-1, endothelin-1; NADPH, nicotinamide adenine dinucleotide phosphate hydrogen; PKC, protein kinase C; ROS, reactive oxygen species; TGF, transforming growth factor; VEGF, vascular endothelial growth factors; FGF, fibroblast growth factor.

The pathogenesis of DN is complex and multifactorial (Figure 1). The metabolic disturbances of diabetes together with hyperglycemia, hyperlipidemia, and hypertension alter the endocrine homeostasis of the vascular wall with a shift in balance of regulatory systems such as nitric oxide (NO)/reactive oxygen species (ROS), vascular endothelial growth factors (VEGF)/VEGF-R, and angiopoietin/tie-2. These pathological changes are fueled by the toxic breakdown products such as oxidized and glycosylated molecules which in turn bind to specific receptors and impair cellular function further* (RAGE lit)*. One of the initial endothelial cell disturbances is an increase in permeability for albumin and other proteins. Over the years this “endothelial dysfunction” will contribute to hypertension and microinflammation with perivascular damage through the altered release of growth factors and cytokines as well as increased permeability for inflammatory cells and cytokines. Mesangial cells, podocytes, pericytes, and tubular epithelial cells are functionally altered and lead to nephropathy, with more inflammation, epithelial cell damage, and increased fibrosis. Over the years these mechanisms impair renal function by vascular rarefication, interstitial fibrosis, and nephron degeneration. The rate of progression of DN is influenced by complex interactions between genetic predisposition, dietary, and lifestyle factors. The patient's genetic profile is responsible for the cellular response to a metabolic stimulus; that is, some patients show an exaggerated endothelial response compared to others when exposed to the same metabolic stress. This may partially explain the spectrum of pathophysiology in diabetic kidneys. In addition, due to the sheer number of nephrons (>1 million/kidney) it takes 15–20 years for kidney failure and ESRD to develop. It is obvious that this process can either be slowed down by a lessened exposure to vascular risk factors or be enhanced if damaging factors are increased.

## 5. Endothelial Cells and Diabetic Vascular Disease

Alterations of endothelial cells and the vasculature play a central role in the pathogenesis of DN [17–19]. Endothelial cells have a key function in regulating the maintenance of capillary function and possibly its regeneration [20, 21]. They are the first cells to encounter metabolic disturbances, that is, alterations of glucose homeostasis and hemodynamic changes such as increase in blood pressure. Originally, the endothelium was believed to act as a “cellophane wrapper” of the vascular tree, with the main function of selectively regulating permeability of water and electrolytes. We now understand that the endothelium embodies a wider range of homeostatic functions. Endothelial cells have the ability to act both as a sensor of metabolic and hemodynamic stress and as a responder with changes in endothelial cell function. Permeability, coagulation, inflammation, and vascular tone are the major mechanisms regulated by endothelial cells [20, 21]. Under physiological conditions, endothelial cells prevent thrombosis by different anticoagulant and antiplatelet adhesiveness and aggregation mechanisms. One important way in which endothelial cells control the clotting system is by regulating the expression of binding sites for anticoagulant and procoagulant factors in the glycocalyx on the endothelial cell surface. In the quiescent state, endothelial cells maintain blood fluidity by promoting the activity of numerous anticoagulant pathways, including the protein C and protein S pathway. After activation, which can be induced by cytokines and also by metabolic factors such as diabetes or hypertension, the balance of endothelial properties can be shifted in favor of clot formation through the coordinated induction of procoagulant and suppression of anticoagulant mechanisms. Inflammatory cytokines, such as tumor necrosis factor, suppress the formation of thrombomodulin, an endothelial anticoagulant cofactor, and induce the expression of tissue factor, which is an important procoagulant cofactor. This shift of balance between procoagulant and anticoagulant factors does not only influence thrombosis and coagulation in the microvasculature but also contributes, both directly and indirectly, to the inflammatory state of the microvasculature in diabetes.

## 6. Inflammation and Oxidative Stress

The endothelium is also a key player in the inflammatory response. Under healthy conditions the endothelium with its smooth glycocalyx provides a slippery surface where circulating blood cells do not adhere [22]. Leukocytes rolling via specific adhesion molecules on the endothelium represent the initial stage of a multistep process leading to extravasation of white blood cells to sites of inflammation or infection [23]. The recruitment of platelets and leukocytes at sites of vascular injury is a very rapid response and is mediated by the release of preformed components expressed and stored by the endothelium. These storage sites within the endothelial cells include Weibel-Palade bodies. Their major constituents are the multimers of von Willebrand factor and P-selectin, one of the initially important and most active promoters of platelet and leukocyte adhesion. In addition, the endothelial cells release angiopoietin-2 which interferes with its antagonist angiopoietin-1 and leads to endothelial cell activation with an inflammatory response and an increase in permeability [24, 25].

Endothelial cells play an important regulatory role in the circulation as a physical barrier controlling endothelial permeability [19, 22]. Permeability can vary between different vascular beds. For example, in the renal glomerulus, permeability is relatively high while, on the other hand, the blood-brain barrier and the blood retinal barrier are almost impermeable to circulating molecules. In diabetes, the permeability of the endothelium is increased. This pathological change leads to an increase in albumin excretion in the kidney nephron and also to enhanced flow of vasoactive substances into the surrounding tissue of organs such as the heart and brain [22].

Furthermore, endothelial cells are a source of vasoregulatory substances such as endothelium-derived NO and prostacyclin which are released in response to physical stimuli and hormones and induce vascular relaxation to reduce leukocyte adhesion and inhibit platelet function [25].

A disturbance of these endothelium-dependent regulatory systems plays an important role in the development of diabetic complications such as DN. Endothelial dysfunction is characterized by a shift in the actions of the endothelium towards reduced vasodilation, a proinflammatory and prothrombotic state. It occurs early in association with the metabolic syndrome but is also influenced by other risk factors such as smoking and hypertension. It appears physical inactivity may also influence endothelial cell function [26].

Endothelial dysfunction is prominent in hypertension and diabetes and may be important at different stages in the development of coronary artery disease, chronic heart failure, peripheral vascular disease, stroke, and chronic kidney failure. Microinflammation and disturbances of endothelial cell permeability are early signs of organ deterioration. Patients who develop diabetes usually consume a high-calorie diet rich in macronutrients which is able to induce vascular abnormalities. It has been shown that high-fat meals may lead to impaired endothelium-dependent vasodilation. Protein, lipid, and glucose loads are associated with a marked production of ROS and oxidative stress [27, 28]. In addition, diabetes is frequently associated with other known cardiovascular risk factors, including hypertension, obesity, and dyslipidemia. All these mechanisms aggravate endothelial dysfunction with increased permeability and low-grade systemic inflammation.

## 7. Vascular Endothelial Growth Factors and Diabetic Nephropathy

The complex relationship between endothelial cells, podocytes, and the VEGF/VEGF receptor system provides an important insight into the intricate network which is responsible for the proper functioning of the renal endothelium under healthy conditions and contributes to the pathogenesis of endothelial cell dysfunction in DN. VEGF-A is expressed by glomerular podocytes in large quantities [29]. The molecule acts by binding to its receptor (VEGF-R2) [30]. Interestingly, this receptor is abundantly expressed on glomerular endothelial cells [31]. VEGF-A crosses the barrier of the basement membrane and the cellular glycocalyx to bind to endothelial cells. Endothelial cells need VEGF-A for maintenance of their various functions (differentiation, permeability, and expression of NOS) and for their survival [31]. Sivaskandarajah et al. convincingly showed that it is deleterious for endothelial cells not to have enough VEGF-A leading to endothelial cell death, loss of glomerular integrity, and glomerulosclerosis [32, 33]. However, too much of VEGF-A also induces endothelial cell damage, an increase in glomerular permeability and inflammation [34]. An appropriate balance of VEGF expression, binding, and signaling is necessary to keep glomeruli healthy and prevent glomerulosclerosis and rarefication. Under diabetic conditions this balance is impaired [35]. Early in the disease the expression of VEGF-A is enhanced and leads to endothelial cell dysfunction [36]. The VEGF system is delicately balanced by regulated expression of VEGF splicing variants [37] and regulated binding to its receptor. The VEGF-A is transported to the VEGF-R2 by specific ligand binding to glycosaminoglycans, providing both storage and adherence in the endothelial cell glycocalyx [38]. This complex of VEGF/heparan sulfates and possible other cofactors is presented to the VEGF-receptors where specific signal transduction is induced and cellular response manufactured. However, also the cellular response is dependent on the availability of effector molecules for the response mechanisms. This additional regulating mechanism has been well studied for the role of eNOS and VEGF [36]. The reduced NO response despite high concentrations of VEGF in the renal vasculature has been called “uncoupling” of eNOS and VEGF, resulting in an impaired cellular response and enhanced pathophysiology in diabetic nephropathy [30]. We suggest that CaD interferes with this mechanism by inhibiting the binding of VEGF (and other growth factors such as FGF) to heparin sulfates, thereby reducing the affinity of the VEGF/HS complex for receptor binding bodies. Such a mechanism would lead to a reduction of VEGF activity in states of enhanced VEGF expression or increased HS sulfate availability. It is also possible that other partners in this signaling complex would be inhibited by CaD. At the same time this modulating effect would not lead to a direct inhibition of VEGF signaling, thereby reducing the side effects observed by direct blockade of the VEGF receptor by antibodies.

The VEGF/VEGF-R system is not the only system where a cellular cross-talk, isoform-specific gene expression, directed molecular transport, and regulated binding and signaling form a complex network for the regulation of a physiological system. In the glomerulus the endothelin system [39] and the angiopoietin/tie-2–system have been characterized. However, in the pathogenesis of DN, the VEGF/VEGF-R system seems to be of critical importance [40]. It will be interesting to see whether novel therapeutic approaches will interfere with these mechanisms.

## 8. Therapeutic Strategies for the Treatment of Diabetic Nephropathy

The complex pathogenesis of DN makes the development of evidence-based therapeutic strategies difficult [41]. Two large studies, the Diabetes Control and Complications Trial conducted in type 1 DM and the United Kingdom Prospective Diabetes Study in type 2 DM, showed that intensive blood glucose control delays the onset and the progression of diabetic microvascular complications including nephropathy [42–44]. These studies strongly suggest that hyperglycemia, that is, the increase in blood glucose concentration, is a major factor in the pathogenesis of DN. However, treatment of hyperglycemia alone does not halt DN progression. In addition to hyperglycemia, enhanced activation of the RAAS is thought to contribute significantly to the pathogenesis of DN. Inhibitors of the RAAS have been shown to lower blood pressure and decrease albuminuria which leads to additionally protective renal and cardiovascular effects [45–47]. Although this approach has shown significant therapeutic effects on the diabetic kidney, it has still made only a small contribution to prevention of the disease [46].

Novel well-tolerated long term treatment strategies which work in the early stages of the diabetes-induced renal changes are urgently needed for preventing or delaying DN. These therapeutic regimens have to be directed at key pathogenic mechanisms and be well tolerated in the long term.

## 9. CaD and Diabetic Microvascular Disease

Calcium dobesilate (CaD) is a synthetic vasoactive drug indicated in the treatment of microangiopathies, in particular diabetic retinopathy. It is the calcium salt of 2,5-dihydroxybenzenesulfonic acid. CaD has been shown to reduce endothelial damage by acting via multiple pathogenic pathways involved in the progression of diabetic microvascular complications [48–51]. The extensive clinical experience with CaD and its favorable benefit/risk profile makes it a promising candidate for the treatment of early stages of diabetic kidney disease.

CaD has been used for more than 40 years in the treatment of diabetic vascular complications, showing consistent efficacy [48–51]. Nevertheless, the mechanism responsible for this effect has not been clearly delineated. CaD has been considered as an angioprotective agent and has been shown to decrease blood viscosity and vascular permeability in patients with diabetic retinopathy [49]. In addition, it has been reported to possess antioxidant capacity that could account for reduction of vascular permeability [50]

Through its unique, multitarget mode of action, CaD has interestingly shown multifaceted pharmacological effects in reducing diabetic microvascular complications. Several studies have demonstrated the effects of CaD on endothelial dysfunction, microinflammation, vasoconstriction, and hyperpermeability. Treatment strategies that include CaD may be useful for preventing or delaying the progression of diabetic retinopathy and nephropathy.

## 10. CaD and Endothelial Cell Function

A reduction in endothelium-dependent relaxation of diabetic vasculature has been associated with both a decrease in NO-mediated vasodilation and an increase in ROS production [48]. Superoxide (O_2_^−^) generation by dysfunctional mitochondria in diabetes has been postulated as the primary initiating event in the development of diabetic complications [52]. The endothelium under diabetic conditions seems to be more sensitive to free radical-induced injury. The abnormal endothelium-dependent relaxation in aorta from diabetic rabbits was restored to normal by superoxide dismutase, suggesting a role for superoxide anions in the endothelial cell abnormality caused by DM [52]. Diabetes-induced vascular endothelial dysfunction can reduce NO production and elevate endothelin-1 (ET-1) levels, diminishing capillary diameter and restricting blood flow. In addition to its direct vasoconstrictor effects, elevated ET-1 may in turn contribute to endothelial dysfunction by inhibiting NO production. CaD exerts strong antioxidative effects in vitro [53]. Ruiz et al. showed that CaD enhanced the endothelium-dependent relaxation induced by acetylcholine in rabbit isolated aorta artery [54]. The effect was clearly endothelium-dependent, indicating that CaD may act on the endothelial derived relaxing factor, NO. A confirmation of these findings came from Suschek et al. In cell cultures obtained from normal Wistar rats and BB rats (a model of type 1 DM), CaD induced a dose dependent increase of NO synthase activity [48]. Additional studies both in vitro and in animal models have confirmed this positive effect of CaD [48, 53, 55].

## 11. eNOS Uncoupling

An enhancement of endothelial permeability is one of the first pathological changes in diabetic microvasculature disease. In vivo, an increase in capillary permeability can be induced by the intraperitoneal administration of prooxidants in rats and measured relative to the concentration in the peritoneal cavity of a dye (Evans blue) injected intravenously. CaD (100 mg/kg orally for 7 days) produced a significant reduction in capillary permeability demonstrating the ability to protect the peritoneal wall from oxidative insult and therefore decrease the induced permeability [51, 56].

CaD has also been shown to reduce the adhesion of inflammatory cells to surfaces [57] and exhibit several anti-inflammatory effects, including the inhibition of NF-KB- and p38 MAPK-mediated leukocyte adhesion to retinal vessels and reduction of adhesion molecules such as ICAM-1 [51]. In the same animal model, CaD showed that reduction of a proinflammatory state and leukocytes recruitment lead to a significant inhibition of tight junction alterations thus protecting the inner blood retinal barrier.

## 12. CaD and FGF/VEGF Inhibition

**Figure 2:**
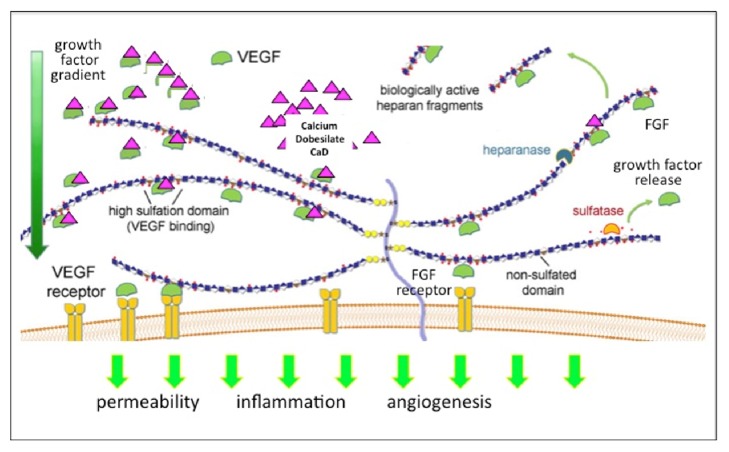
The FGF/VEGF system and its alterations in diabetes: the effects of CaD. In diabetic patients VEGF and its receptors are overexpressed. Circulating growth factors requires two binding sites in order to elicit a cellular response: (1) to heparan sulfate domains on extracellular proteoglycans and (2) to its specific membrane-bound receptor. The heparan sulfate domains provide a gradient for growth factors and allow coordinated binding to their receptors where intracellular pathways are activated which lead to endothelial dysfunction, albuminuria, and angiogenesis. The heparin binding sites are regulated physiologically by heparanases and sulfatases. CaD binds specifically to the negatively charged domain of growth factors thereby interfering with their binding to their receptors and thus reducing endothelial cell dysfunction, albuminuria, and angiogenesis under diabetic conditions. While VEGF antibodies completely block the effects of VEGF on the intracellular signaling pathways and thereby also blocking the VEGF-induced signals which are necessary for endothelial cell survival, interference with the heparin sulfate binding sites reduces the binding of VEGF and FGF to its coreceptor and therefore reduces its effects on endothelial cells but does not abolish the effect of VEGF on its specific membrane-bound receptor. VEGF, vascular endothelial growth factor; FGF, fibroblast growth factor.

Vascular endothelial growth factor is a key factor in the pathogenesis of diabetic microvascular disease by inducing angiogenesis and vascular permeability. CaD has shown antiangiogenic activity and reduces vascular permeability under diabetic conditions [58, 59]. CaD significantly inhibited the proliferation of human umbilical vein endothelial cells (HUVEC) induced by VEGF (10 ng/ml), without significantly affecting HUVEC proliferation in the absence of VEGF and reduced also VEGF-induced angiogenesis in vivo [58]. Previous investigators had demonstrated that CaD interferes with heparin binding on fibroblast growth factor (FGF) and inhibits the signaling of FGF via its receptors FGFRs [60]. These experiments have demonstrated that CaD recognizes both growth factors, as a heparin antagonist within the binding site in these polypeptides, changes the three-dimensional structure of the growth factor at their receptor recognizing site, and is capable of dissociating the receptor-growth factor signaling complex. CaD seems therefore to be a member of a novel group of molecules which inhibit growth factors not only by interfering with their receptor binding but by regulating growth factor activity by specifically interfering with its interaction with glycoproteins. This mechanism explains not only the inhibitory effect on FGF and VEGF by CaD but also the low rate of side effects as compared to VEGF antibodies in diabetes [60, 61]. While VEGF antibodies completely block the effects of VEGF on the intracellular signaling pathways and thereby also block the VEGF-induced signals which are necessary for endothelial cell survival, interference with the heparin sulfate binding sites reduces the binding of VEGF and FGF to its coreceptor and therefore reduces its effects on endothelial cells but does not abolish the effect of VEGF on its specific membrane-bound receptor (Figure 2).

## 13. CaD and Oxidative Stress

Increases in oxidant production clearly have been shown to occur when vascular or glomerular cells are exposed to hyperglycemia. The metabolism of glucose via mitochondrial pathways and the activation of NADPH oxidases via PKC activation have been shown to contribute significantly to oxygen radical production. CaD has been described as having strong antioxidative effects both in vitro and in vivo. CaD has proven to be an oxygen free radical scavenger and to inhibit free radicals production both in vitro and in vivo animal models. In vitro, CaD has been shown to scavenge oxygen free radicals generated by reaction between xanthine and xanthine oxidase in absence or presence of DMSO (dimethylsulphoxide) and iron chloride (FeCl_2_) in a dose dependent manner (human polymorphonuclear blood cells). Therefore it is believed to inhibit both oxidative damage in capillary cells and inflammatory cascades [28–32].

Increasing evidence indicates that the disruption of mitochondrial bioenergetics may be important in the development and progression of diabetic nephropathy. As the kidney relies on oxidative phosphorylation to provide the ATP for tubular reabsorption it is not surprising that mitochondrial homeostasis is strictly essential for an optimally functioning kidney. There is ample evidence that there is a disturbance of mitochondrial bioenergetics in the diabetic kidney.

## 14. CaD and Diabetic Nephropathy

Dong and coworkers have analyzed the effects of CaD on the expression of glomerular tissue inhibitor of metalloproteinase 1 (TIMP1), collagen IV, and ultrastructure of glomerular basement membrane in diabetic STZ rats [62, 63]. Their results showed that, after 12 weeks, kidney function in CaD-treated animals increased and was significantly improved compared to that in control diabetic animals. Electron microscopy showed that thickness of glomerular capillary basement membrane (GBM) was also improved. Podocyte foot processes were preserved and expression of TIMP1 and collagen IV were significantly less in treated rats. CaD may improve DN by inhibiting the overaccumulation of collagen IV and TIMP1. Recently Jafarey et al. showed that CaD is effective in protecting rats against gentamicin-induced nephrotoxicity. This protective effect of CaD has been considered probably dependent on its antioxidant properties [64].

Zhang and colleagues analyzed the effects of CaD in diabetic patients [65]. A total of 121 patients with type 2 DM and albuminuria received CaD (500 mg, 3 times/day) for 1, 2, or 3 months, respectively. Urinary albumin excretion, medium and low shear rate, and whole blood viscosity were significantly lower in the treated patients. The rate of microalbuminuria normalization after 12 weeks was 90%. In addition, the benefit was positively correlated with CaD treatment duration. They additionally measured the plasma concentrations of plasminogen inhibitor-1 (PAI-1) and described a decrease of PAI-1 during CaD treatment, which contributes to the therapeutic effect of CaD. Other positive effects of CaD, such as a decrease in endothelin, may be related to the molecular effects of the drug.

The specific mode of action of CaD, as compared to other antiproteinuric drugs such as RAAS blockade, has been demonstrated in the study by Dong et al. [66]. Patients were randomly assigned to three groups: placebo, CaD, and the angiotensin converting enzyme- (ACE-) inhibitor perindopril. There was a comparable decrease in albuminuria in the two treatment groups. However, a comparison of different markers of endothelial dysfunction such as endothelin and NO showed a clear difference in the action of CaD versus the ACE-inhibitor. In addition to decreasing PAI-1, CaD also reduced endothelin levels and increased NO. In other studies, in hemodialysis patients and in patients affected by early stages of DN, reduced serum levels of high-sensitivity C-reactive protein, improved micro-inflammatory state, decreased serum levels of ET-1, and increased levels of NO were observed after CaD administration [63, 67].

The hypothesis of additive effects of CaD to a standard therapy of RAAS blockade was analyzed in four smaller studies. In all four studies, an additional decrease in albuminuria after the combination treatment with CaD was observed when combined with benazepril, enalapril, perindopril, or losartan [68–71].

## 15. Meta-Analysis of CaD Treatment on Diabetic Nephropathy in Chinese Patients with Type 2 Diabetes

Meta-analysis of the data obtained in Chinese diabetic patients with albuminuria from seven studies [63, 66, 68–70, 72, 73] supports the hypothesis that CaD may reduce albuminuria in these patients and is a promising therapeutic strategy for microvascular disease not only in the retina but also in the kidney (Tables 1 and 2).

These seven studies were identified by a literature search of MEDLINE, EMBASE, and CENTRAL from the time recording commenced until December 2015. The three study selection criteria used were (1) randomized placebo-controlled trials; (2) study duration ≥ 12 weeks; and (3) treatment of Chinese patients with type 2 diabetic nephropathy. The eligibility of all studies retrieved from the databases was screened independently by two reviewers based on those three predetermined selection criteria. Disagreements between reviewers were resolved by consultation with a third investigator. A validated 3-item scale was used to evaluate the overall reporting quality of the trials selected for inclusion in the meta-analysis. The meta-analysis was performed by computing the weighted mean difference (WMD) and 95% confidence interval (CI) for change from baseline to study endpoint for CaD versus control treatment groups. All statistical analyses were performed with the Review Manager statistical software package (Version 5.2).

**Figure 3:**
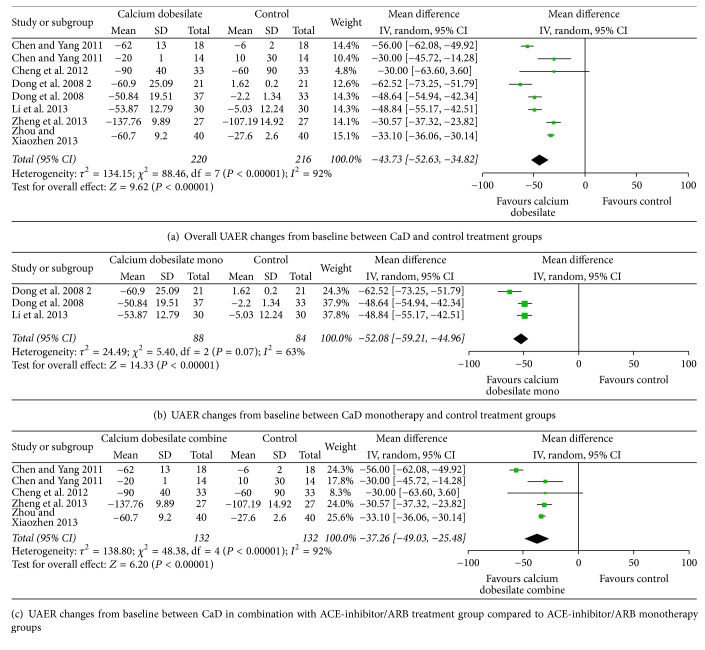


Treatment with CaD was received by 220 patients while a control treatment group with placebo with standard diabetic treatment was received by 216 patients. Overall, when compared to the control group, CaD treatment, either as monotherapy or in combination with ACE-inhibitor/angiotensin II receptor blocker (ARB), led to a significantly greater change from baseline in urine albumin excretion rate (UAER) (WMD, −43.73 mg/24 h; 95% CI, −52.63 to −34.82 mg/24 h, *p* < 0.001) (Figure 3(a)). As monotherapy only, CaD was associated with a significantly greater change in UAER (WMD, −52.08 mg/24 h; 95% CI, −59.21 to −44.96 mg/24 h, *p* < 0.001) (Figure 3(b)). When comparing CaD in combination with ACE-inhibitor/ARB to control treatment (ACE-inhibitor/ARB alone), the combination treatment was associated with a significantly greater change in UAER (WMD, −37.26 mg/24 h; 95% CI, −49.03 to −25.48 mg/24 h, *p* < 0.001) (Figure 3(c)).

## 16. Conclusion

New therapeutic strategies in DN need to be specifically targeted towards the pathophysiology of the disease. The earlier these therapeutic strategies can bring about positive effects on vascular changes and prevent the vasculature in diabetics from deteriorating, the better we will be able to preserve renal function in patients with diabetes. Studies evaluating anti-inflammatory and antioxidative strategies in DN demonstrate the need and value of these novel treatment avenues.

CaD is an established vasoactive and angioprotective drug that has shown a unique, multitarget mode of action in several experimental studies and in different animal models of diabetic microvascular complications. At the molecular level, CaD reduces oxidative stress and inhibits growth factors such as FGF and VEGF. Importantly, CaD has shown to be effective in the treatment of diabetic retinopathy (mainly type 2 DM).

Even though more evidence is needed to better understand the role of CaD in other diabetes-related microangiopathies such as DN, recent findings have demonstrated a strong rationale for its use in reducing UAER and markers of inflammation as well as improving endothelial function. Its effects on VEGF and on oxidative stress make it an attractive therapeutic compound especially in the early stages of the disease. These findings, although promising, need further confirmation in prospective clinical trials with CaD.

## Figures and Tables

**Table 1:** Baseline demographic and disease characteristics of patients included in meta-analysis.

	CaD	Control
*N*	220	216
Age (years)	59.3 ± 6.0	58.3 ± 7.1
Male (%)	41.5%	45%
DM duration (year)	8.1 ± 2.1	7.9 ± 2.0
Baseline HbA1c (%)	7.0 ± 0.7	7.2 ± 1.0

**Table 2:** Comparisons between CaD treatment and control treatment groups in UAER changes from baseline.

	Number of studies	Number of subjects (CaD versus control)	WMD from baseline	95% CI	*I* ^2^
All	7	220/216	−43.73^*∗*^	−52.63, −34.82	92%
Monotherapy	3	88/84	−52.08^*∗*^	−59.21, −44.96	63%
Combination with ACEI/ARB treatment	4	132/132	−37.26^*∗*^	−49.03, −25.48	92%

^*∗*^ *P* < 0.001.
